# Inequities in Care During Pregnancy Loss: Empirical Insights From Experiences With Canadian Perinatal Care

**DOI:** 10.1111/birt.70020

**Published:** 2025-09-30

**Authors:** Wendy A. Hall, Nisha Malhotra, Esther Clark, Karen Hodge, Gabrielle Griffith, Saraswathi Vedam

**Affiliations:** ^1^ University of British Columbia School of Nursing Vancouver British Columbia Canada; ^2^ Midwifery, Faculty of Medicine University of British Columbia Vancouver British Columbia Canada; ^3^ Alberta Health Services Edmonton Alberta Canada; ^4^ Community Steering Council Adaptability Counselling and Consultation Vancouver British Columbia Canada

**Keywords:** perinatal loss, racial disparities, respectful maternity care

## Abstract

**Background:**

Individuals experiencing perinatal loss are entitled to respectful maternity care, but a paucity of research examines respectful care at the time of pregnancy loss.

**Method:**

We used data from an online cross‐sectional survey (July 2020–February 2022), where 172 individuals reported on early (miscarriage) and late (late second trimester, stillbirth, neonatal death) losses since 2009. We aimed to explore inequities in respectful care experiences among individuals experiencing a late versus early perinatal loss in Canada. We assessed their experiences using the Mothers' Autonomy in Decision Making (MADM) scale and the Mothers on Respect Index (MORi). We created the Compassionate Disclosure of (perinatal) Loss (CDL) index to measure respectful care at the time of a loss. A single separate item, provider not listening to the individual's expression of concerns during pregnancy, was also analyzed.

**Results:**

The early and late loss groups differed in education levels. Individuals who self‐identified as Indigenous/Black/People of Color (IBPOC) had lower odds of scoring in the top quartile on MADM and MORi scales (AOR = 0.31, 95% CI 0.13, 0.75; AOR = 0.34, 95% CI 0.13, 0.86); and higher odds of reporting that providers did not listen to their concerns prior to the loss (AOR = 2.61, 95% CI 1.24, 5.48). Psychometric analysis supported the CDL index. Participants experiencing late loss had higher odds of reporting top quartile CDL scores than those experiencing early loss (AOR = 3.08, CI 1.22, 7.77).

**Conclusion:**

Canadian individuals with perinatal loss report disproportionately poorer care when they are experiencing a miscarriage and when they identify as IBPOC.

## Graphical abstract

Inequities in Canadian Care during Pregnancy Loss.

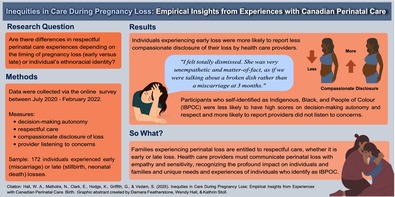

## 1 Introduction

Perinatal loss is the non‐voluntary end of pregnancy or death of a baby from conception to 28 days of neonatal life; it includes miscarriage, stillbirth/fetal death, and neonatal death [1, 2, 3]. Miscarriage is defined as the spontaneous death of an embryo or fetus prior to 20 weeks of gestation [4]. Although it is difficult to quantify the number of miscarriages in Canada, a recent paper from British Columbia reported 15,000 per annum [4]. In 2022, there were 3165 fetal deaths (stillbirths) in Canada, where the fetus was at 20 weeks or more gestation, resulting in a fetal death rate of 8.9 per 1000 total births [5]. There were 3.5 neonatal deaths per thousand live births [6]. Perinatal loss is a devastating experience serving as a major source of stress for individuals and a form of reproductive trauma [1, 2, 3]. Respectful perinatal bereavement care has important implications for the well‐being of parents as well as healthcare professionals [7, 8]. Shorey et al. conducted a scoping review of studies on the impact of bereavement on health care professionals. They concluded that in high‐resource countries there is a need to provide institutional support and culturally sensitive education and training so that professionals can provide consistent and timely, interdisciplinary, compassionate care for families suffering a perinatal loss [9].

Respectful maternity care maintains the dignity, privacy, and confidentiality of pregnant and birthing individuals, is free from harm and mistreatment, and enables informed choice and continuous support during labour and birth [10, 11]. Access to safe, high‐quality reproductive health care, particularly responsive, supportive pregnancy care, can dramatically reduce global rates of maternal morbidity and mortality [10].

Very little is known about experiences of respectful perinatal care in Canada, including for those who experience pregnancy loss. In one recent provincial study in Ontario, people with a history of pregnancy loss reported where they received care and who provided support (provider, peer or community support). The items did not capture detailed information about interactions with providers. The authors used thematic analysis to conclude that individuals were inadequately informed, supported, and cared for by healthcare professionals who lacked necessary skills, and that experiencing stigma from providers exacerbated their loss experience [12]. In addition, there has been a lack of attention to the experiences of loss among communities that often experience stigmatization and marginalization [13]. In the current analysis, we examine detailed mixed‐methods data on the characteristics of respectful care among people who experience perinatal loss across Canada, including experiences for persons from diverse population groups.

## 2 Methods

The data in this analysis are derived from the RESPCCT Study, a Canadian online cross‐sectional survey distributed from July 2020 to February 2022 to evaluate individuals' perinatal service experiences. With the support of the Canadian Institutes of Health Research (2018–2023), our team conducted a national study that enabled a comprehensive analysis of experiences and outcomes of respectful care. We used a community participatory action research (CPAR) approach to design an instrument that measured patient‐oriented outcomes of perinatal care across Canada, with a focus on interactions between service users, service providers, and health systems. A Community Steering Council actively engaged throughout the study by guiding decisions regarding study design, item selection, recruitment, data collection, and analysis. The Council represented a diverse range of lived experiences and perspectives. The survey was translated into seven languages and mounted onto the Qualtrics platform. A survey link was included in the invitation email to prospective participants and advertisement shared on social media. Ethics approval for this study was obtained from the British Columbia Behavioral Research Ethics Board (H18‐01961). Participants completed click‐through online consent forms before beginning the survey [14].

The authors of the current paper position themselves as clinicians, parents, and researchers with extensive exposure to perinatal loss. A detailed description of the methods including development of study measures, content validation, and recruitment is published elsewhere [14].

### 2.1 Survey Instrument

A multi‐stakeholder team comprising the Steering Council, NGO leaders, researchers, and knowledge users collaborated to develop the survey [14, 15]. The RESPCCT survey instrument includes 210 validated patient‐designed outcome measures that assess experiences of autonomy, respect, disrespect, stigma, discrimination, and mistreatment; responsiveness of healthcare providers; choice of care options; verbal and non‐verbal communication; patient reactions to experiences of care; and health systems factors that are particularly resonant with marginalized populations. Logic branching enabled participants with certain characteristics (e.g., loss) to provide additional relevant details.

### 2.2 Sample

The survey provided an opportunity for individuals who had encountered pregnancy loss (*n* = 172) in Canada, within the 10 years preceding data collection, to respond to questions about their care. We excluded those who reported a planned abortion (*n* = 54). Participants from all provinces and territories across Canada were recruited, including historically underrepresented populations. We divided the respondents into a miscarriage and late loss group. The miscarriage and late (2nd‐trimester loss post‐viability, stillbirth, neonatal death) perinatal loss groups were based on literature that defines miscarriage as occurring prior to 20 weeks gestation [4] and other literature indicating that care providers' approach, language, and perception of trauma are influenced by the fetal stage of development [16].

### 2.3 Study Measures

In this study, we examined four care experiences: (1) autonomy in decision‐making (7 items), (2) respectful care (7 items), (3) care providers' listening to maternal concerns leading up to a loss (1 item), and (4) providers' compassionate disclosure of perinatal loss (6 items).

We utilized validated instruments to assess the first two care experiences. The autonomy of individuals was gauged using the MADM (Mother's Autonomy in Decision Making) scale [17]. The MADM encompasses seven items (See Appendix A). Respondents rated these items on a scale ranging from 1 (Completely disagree) to 6 (Completely agree). Higher scores mean that childbearing people were able to exercise more autonomy in decision‐making. For the full sample, the MADM index demonstrated high internal consistency, with a Cronbach's alpha coefficient of 0.947. The mean score for autonomy was 26.14, with a range of 7–42. Respectful care was evaluated through the MORi (Mothers on Respect Index), which consists of seven items (See Appendix A) and exhibited a Cronbach's alpha of 0.918 [18]. The mean score for respectful care was 29.65, ranging between 7 and 42. Higher scores mean that childbearing people experienced more respectful care. Previous research has supported the feasibility, internal consistency, and validity of these tools for assessing respect and autonomy in maternity care [19].

**FIGURE 1:**
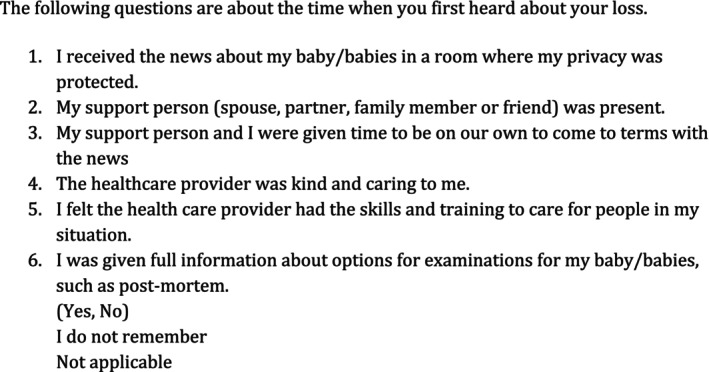
Questions for the Compassionate Disclosure of Loss (CDL) index.

To address the need for a comprehensive assessment of respectful care during perinatal loss, we created the Compassionate Disclosure of Loss (CDL) index. This index aimed to capture respectful care for families experiencing loss by using six items to assess individuals' experiences when they first receive the news of perinatal loss. The items are introduced with a single sentence: The following questions are about the time when you first heard about your loss. The items for the CDL refer to: (i) protection of privacy, (ii) the presence of a support person, (iii) time to come to terms with the loss, (iv) kind and caring provider engagement, (v) adequate provider skills and training, and (vi) full information on options for examination of the baby (See Figure 1). Each item is represented as a dichotomous variable, with 1 indicating agreement and 0 indicating disagreement. For analysis of the CDL, we added the ‘do not remember’ response to the ‘no’ response for negative events. We argue that, when respondents perceived events negatively, they would be unlikely to indicate they did not remember them. CDL scores ranged from 0 to 7; the mean score for the CDL index was 3.69. Higher scores mean that childbearing people reported more compassionate/respectful communication following their loss. Using Cronbach's alpha, the internal consistency of the CDL measure was 0.80. A factor analysis on the CDL index revealed a single factor with an eigenvalue of 3.07009, explaining 51.17% of the total variance. The factor loadings for each item ranged from 0.4806 to 0.8100, indicating that all six items measure a single underlying construct of compassionate disclosure. The likelihood ratio test (χ^2^ = 274.58, *p* < 0.0000) confirmed that the factor model fit the data. These findings support the unidimensional nature of the CDL index, indicating it is a valid measure of compassionate disclosure following perinatal loss.

Participant perceptions of care providers listening to concerns during their pregnancies, before they experienced a loss, were measured in response to one item on the survey: “I felt my health care provider(s) did not listen to my concerns leading up to the loss”. The item permitted a yes or no response. The items in the CDL index indicate how news of the loss was conveyed to the parents (See Appendix A). In addition to responding to the survey scales and items, respondents were invited to share their experiences in open text boxes.

### 2.4 Analysis

The analysis incorporated confidence intervals and *p*‐values from pooled *t*‐tests and Pearson chi‐square tests to evaluate differences in means and frequencies. Multivariable logistic regression analysis was employed to investigate the influence of various factors and individual characteristics on an individual's pregnancy loss care experience. STATA SE 18 statistical software [20] was used to analyze data. Because the scales and indices (MADM, MORi, CDL) were constructed from multiple items—some of which are more relevant at different stages of pregnancy loss—adjustment for timing of loss (early versus late) was included as a binary variable in all analyses.

The scores obtained from the three indices were used to generate three binary variables: top quartile MADM, top quartile MORi, and top quartile CDL. If the score fell within the top 25% of the respective index, these variables were assigned a value of 1. The top MADM quartile variable denotes a high level of autonomy in decision‐making, while the top MORi quartile variable signifies a high level of respectful care. The top CDL quartile indicates more compassionate disclosure of news of perinatal loss.

The researchers employed multivariable logistic regression, which allows examination of how factors—such as racial identity, education, sexual minority status, income, place of residence, and timing of pregnancy loss—were associated with experiences of care, while taking into account the influence of all other variables simultaneously. To more precisely distinguish the effect of race on care experiences, we adjusted for the influence of other social and contextual variables on experiences of care. Including education level recognized its influence on individuals' engagement with healthcare providers and advocacy for their care. The inclusion of sexual minority status reflected potential encounters with unique barriers or biases within healthcare systems. Income adequacy was a factor because financial resources can affect access to healthcare and service quality. Including place of residence acknowledged differences in healthcare options, provider training, and available supports across rural, urban, and large city settings. Variability in provider attitudes and care practices arising from the stage of pregnancy loss required the creation of a binary variable for timing of loss (early versus late [after 20 weeks of pregnancy]). The year of pregnancy accounted for temporal changes in healthcare policy or practice, including those related to the COVID‐19 pandemic.

For our analysis, Indigenous or racialized identity versus white was categorized as Indigenous, Black, or Person of Color. Specifically, individuals identifying as Indigenous encompassed First Nations, Métis, or Inuk (Inuit), and People of Color included respondents who identified as Central Asian, East Asian, Latinx or Hispanic, Middle Eastern, South Asian, and Southeast Asian. Education level included high school or less, college completion, and graduate or professional degree. Sexual minority status (those who did not select heterosexual) included Gay/Lesbian, Bisexual, Queer, Two‐Spirit, Asexual, declined/unsure, or ‘none of the above.’ Income sufficiency was characterized as not enough/enough versus more than enough. Place of residence was indicated by rural and small town, a city, and, if so, city size. Because data spanned up to 10 years preceding the survey and encompassed the COVID‐19 lockdown period, the analysis incorporated a year trend to account for temporal changes in care quality. Segments from open‐text boxes were included to enrich the quantitative findings.

## 3 Results

Table 1 provides an overview of the demographic characteristics of individuals who experienced early and late pregnancy loss. Among the 172 individuals studied, 58.1% experienced miscarriage (early loss), while 41.9% encountered stillbirth, neonatal loss, or late second‐trimester loss (SB/NB/Late 2T). The mean age for both groups was similar. Indigenous/Black/People of Color participants comprised 30.2% of the sample; white participants accounted for 69.8% of our sample. Regarding education, individuals with graduate or professional degrees were more prevalent among those who experienced early loss (50.5%) compared to late loss (18.1%), *p* < 0.001. Because other characteristics, such as income sufficiency, sexual minority status, and place of residence did not differ significantly between the early loss and late loss groups, we combined the early and late loss groups for the regression analysis.

**TABLE 1:** Descriptive statistics for early and late pregnancy loss.

	Early loss miscarriage	Late loss^c^	Total	Test
	(*n* = 100) (58.1%)	(*n* = 72) (41.9%)	(*n* = 172) (100.0%)	
Age	31.98 (5.14)	31.36 (4.87)	31.72 (5.02)	0.427
Racial identity				
White	68 (68.0%)	52 (72.2%)	120 (69.8%)	0.552
(Indigenous^a^/Black/POC^b^)^d^	32 (32.0%)	20 (27.8%)	52 (30.2%)	
Income to meet needs				
Enough or Not Enough	60 (60.0%)	39 (54.2%)	99 (57.6%)	0.445
More than Enough	40 (40.0%)	33 (45.8%)	73 (42.4%)	
Education				
High School or less	26 (26.8%)	32 (44.4%)	58 (34.3%)	< 0.001
Completed College	22 (22.7%)	27 (37.5%)	49 (29.0%)	
Graduate/Professional degree	49 (50.5%)	13 (18.1%)	62 (36.7%)	
Sexual minority				
Heterosexual	77 (78.6%)	56 (77.8%)	133 (78.2%)	0.901
Sexual Minority	21 (21.4%)	16 (22.2%)	37 (21.8%)	
Place of residence				
Rural/sm. Town (< 30,000)	29 (29.9%)	24 (33.3%)	53 (31.4%)	0.180
City (30,499,000)	31 (32.0%)	30 (41.7%)	61 (36.1%)	
Big City (> 500,000)	37 (38.1%)	18 (25.0%)	55 (32.5%)	

*Note:* Mean (standard deviation): *p*‐value from a pooled *t*‐test. Frequency (Percent %): *p*‐value from a Chi‐square test.

^a^ First Nations, Métis, or Inuk (Inuit).

^b^ Central Asian, East Asian, Latinx or Hispanic, Middle Eastern, South Asian, and South East Asian.

^c^ Stillbirth/newborn/Late 2nd Trimester Loss.

^d^ Includes either Indigenous, Black, or People of Color.

The two groups differed in the care they experienced: a lower percentage of respondents in the early loss group (29%, *p* = 0.001) felt that the news of loss was disclosed to them in a compassionate manner compared to the ‘late loss’ group (40.3%). By illustration, one of the participants shared their experience: “*I felt totally dismissed. She was very unempathetic and matter‐of‐fact, as if we were talking about a broken dish rather than a miscarriage at 3 months*.” The other measures of respectful care, MADM and MORi, did not demonstrate statistically significant differences between those participants with early loss and those with late loss (Table 2).

**TABLE 2:** Respectful care.

	Early loss miscarriage	Late loss^**c**^	Total	Test
	*n* = 100 (58.1%)	*n* = 72 (41.9%)	*n* = 172	
Top quartile MADM				
No	76 (76.0%)	45 (62.5%)	121 (70.3%)	0.056
Yes	24 (24.0%)	27 (37.5%)	51 (29.7%)	
Top quartile MORI				
No	73 (76.8%)	44 (63.8%)	117 (71.3%)	0.068
Yes	22 (23.2%)	25 (36.2%)	47 (28.7%)	
Didn't listen to concern				
No	57 (58.8%)	37 (51.4%)	94 (55.6%)	0.340
Yes	40 (41.2%)	35 (48.6%)	75 (44.4%)	
Top Quartile CDL				
No	66 (84.6%)	38 (61.3%)	104 (74.3%)	0.002
Yes	12 (15.4%)	24 (38.7%)	36 (25.7%)	

*Note:* Frequency (Percent %): *p*‐value from Chi‐square test.

^**c**^ Stillbirth/newborn/Late 2nd Trimester Loss.

The multivariable logistic regression analysis revealed significant associations between demographic variables and several indices, including the top quartile MADM, top quartile MORi, the single item ‘Didn't Listen to Concern,’ and top quartile CDL indices. The “adjusted odds ratio” (AOR) indicated the likelihood of a particular group reporting a specific experience compared to a reference group, after accounting for (or “adjusting for”) differences in other characteristics that were included in the regression. Adjusted odds ratios help separate the unique impact of each factor, making the findings more accurate and relevant to practitioners.

Individuals who identified as Indigenous/Black/Persons of Color exhibited lower odds of reporting autonomy in decision making (AOR = 0.31, 95% CI [0.13, 0.75]) and respectful care (AOR = 0.34, 95% CI [0.13, 0.86]). These groups also had higher odds of reporting instances where concerns were not listened to (AOR = 2.61, 95% CI [1.24, 5.48]). As indicated previously, for open text boxes, respondents were given an option: “If you would like to share more about your experience, please use the space below”. The following quotes describe some experiences these participants had during their losses that exemplify the associations described above (Table 3).

**TABLE 3:** Adjusted odds ratio: factors associated with experiences of care.

Logistic regression	Top quartile MADM	Top quartile MORI	Didn't listen to concerns	Top Quartile CDL
Racial identity				
White	1.00	1.00	1.00	1.00
(Indigenous^a^/Black/POC^b^)^d^	0.31*** [0.13, 0.75]	0.34** [0.13, 0.86]	2.61** [1.24, 5.48]	1.02 [0.41, 2.59]
Sexual minority				
Heterosexual	1.00	1.00	1.00	1.00
Sexual Minority	1.60 [0.66, 3.88]	1.64 [0.64, 4.22]	1.28 [0.55, 2.96]	0.45 [0.12, 1.66]
Age	0.97 [0.89, 1.05]	1.03 [0.95, 1.12]	0.97 [0.90, 1.04]	0.94 [0.85, 1.04]
Income to meet needs				
Enough or Not Enough	1.00	1.00	1.00	1.00
More than Enough	0.84 [0.39, 1.83]	1.39 [0.62, 3.09]	0.61 [0.30, 1.23]	1.61 [0.65, 3.95]
Education				
High School or less	1.00	1.00	1.00	1.00
Completed College	1.54 [0.60, 3.94]	1.11 [0.44, 2.80]	0.81 [0.34, 1.92]	1.38 [0.45, 4.18]
Graduate/Professional degree	0.84 [0.30, 2.34]	0.30** [0.10, 0.90]	1.79 [0.71, 4.47]	0.93 [0.25, 3.41]
Place of Residence				
Rural/sm. Town (< 30,000)	1.00	1.00	1.00	1.00
City (30–499,000)	1.20 [0.46, 3.09]	1.05 [0.42, 2.64]	0.73 [0.32, 1.69]	0.96 [0.35, 2.69]
Big City (> 500,000)	2.59* [0.94, 7.12]	1.26 [0.45, 3.51]	0.55 [0.22, 1.37]	1.91 [0.67, 5.44]
Perinatal Loss				
Early Loss	1.00	1.00	1.00	1.00
Late Loss^c^	1.70 [0.78, 3.73]	1.32 [0.58, 3.01]	1.88* [0.91, 3.85]	3.08** [1.22, 7.77]
Year of Pregnancy	0.91 [0.81, 1.02]	1.00 [0.89, 1.14]	1.06 [0.96, 1.17]	0.97 [0.85, 1.11]
Number of observations	167	159	164	135
Pseudo *R* ^2^	0.0978	0.0927	0.0749	0.0899

*Note:* Values are Adjusted odds ratio; 95% Confidence Interval [CI]; Robust estimators are used.

^a^ First Nations, Métis, or Inuk (Inuit).

^b^ Central Asian, East Asian, Latinx or Hispanic, Middle Eastern, South Asian, and South East Asian.

^c^ Stillbirth/newborn/Late 2nd Trimester Loss.

^d^ Includes either Indigenous, Black, or People of Color.

*** *p* < 0.01.

** *p* < 0.05.

* *p* < 0.1.

One Indigenous participant wrote: *“When I was told I allegedly lost the baby the doctor was extremely cold‐hearted and acted as if it was good news because we were ‘young’* (21) *and probably because we're Indigenous. She gave us barely any time to process before pushing very hard that I HAD to get a d & c. I was never told of any other options for anything related, I was never offered an ultrasound picture to hold onto and was never told or realized I could go somewhere else to be sure of what they told me. Because with how secretive and awful they were to me, and also with the record of that hospital, I struggle every day with if I was lied to or not.”*

One participant, who identified as Middle Eastern and described a pregnancy that resulted in stillbirth, reported that health care providers did not listen to her concerns, and wrote: “*Only sometimes. I felt my baby kept having hiccups. I was concerned about that. I was 39 weeks and measuring 42[cm] due to extra fluid, and I was in pain and just wanted to be induced, and my doctor declined and said no need to induce unless you go past due date. After the loss, she said, ‘I wish we induced you.”*

Regarding sexual minority status, the odds ratios for the top quartile MADM, MORi, and CDL indices were 1.60, 1.64, and 1.28, respectively; however, those associations were not statistically significant. A significant association was identified between individuals residing in big cities (> 500,000) having higher odds of being in the top quartile for the CDL index (AOR 1.91, *p < 0.1).

Experiencing late second trimester, stillbirth, or neonatal loss was associated with higher odds of being in the top quartile for the CDL index (AOR 3.08, **p < 0.05), meaning that individuals experiencing late pregnancy loss reported relatively more compassionate disclosure of their perinatal loss than those with early pregnancy loss.

## 4 Discussion

This study, using Canadian online cross‐sectional survey data, assessed whether participants experienced respectful and compassionate care during a perinatal loss experience. Our aim was to include diverse and often underrepresented groups in our study. This study explored four care experiences for individuals reporting a perinatal loss in the previous 10 years: (1) autonomy in decision‐making, (2) respectful care, (3) care providers listening to concerns leading up to a loss, and (4) providers' compassionate disclosure of perinatal loss.

The demographic characteristics of our sample suggest some success for including traditionally underrepresented participants. Our sample consisted of 30.2% identifying as either Black, Indigenous, or People of Color. Those proportions fit with Statistics Canada's [21, 22] data, which indicated that 30.8% of Canadians identify as Black/Indigenous/People of Color. Additionally, 57.6% of our sample reported only enough or ‘not enough’ income to meet their needs, and 34.3% reported high school completion.

The study results indicated that participants reporting a miscarriage were more likely to have graduate and/or professional degrees than the participants reporting a late loss. The literature provides some support for this finding. A study of almost 80,000 Manitoban women reporting a first miscarriage indicated that women experiencing a first miscarriage compared with those experiencing a singleton live birth were more likely to be nulliparous and older [23]. Obtaining post‐secondary education has the potential to delay childbearing.

The finding that participants residing in big cities had higher odds of being in the top quartile for the CDL index is not surprising. A global scoping review of the needs and experiences of healthcare professionals facing perinatal death found that 18 of the 28 studies they reviewed reported that healthcare professionals wanted formal training from hospitals about culturally specific bereavement care and communication skills [9]. Hospitals in larger centers are more likely to have higher patient volumes, more exposure to individuals experiencing perinatal loss, resources to support formal training about care and communication skills, and higher staffing numbers.

We found that respondents in the miscarriage group were less likely to regard their losses as being disclosed to them in a compassionate manner. Hiefner and Villreal point out that some healthcare providers fail to understand and validate the meaning and significance of miscarriage loss [16]. Consequently, Hiefner and Villreal proposed an integrated behavioral model of care to respond to the individuals experiencing disenfranchisement of their loss. A disenfranchised loss is captured by the lack of open acknowledgement, public mourning, and/or social support provision. Disenfranchising perinatal loss interferes with individuals' grieving.

In our study, individuals who identified as Indigenous/Black/Persons of Color had lower odds of experiencing autonomy (MADM) and respectful care (MORi) and higher odds of reporting that healthcare providers did not hear their concerns. These findings are very concerning but not surprising. Previous work undertaken in British Columbia using the MADM index indicated autonomy was significantly lower among racialized participants and when they held back questions because care providers were rushed and/or they perceived poor treatment due to their race/ethnicity [17]. Moreover, the *In Plain Sight* report generated in British Columbia from surveys provided by almost 3000 Indigenous people documented participants' perceptions of poorer service (23%), being treated as ‘bad’ parents (14%), never being included in care decisions (11%), and not feeling like their needs were taken seriously (27%) [9].

We developed the CDL index to assist with our understanding of the components of perinatal loss. It is an important contribution to quality improvement initiatives because the moment of the diagnosis of the loss has been identified as a critical time for families who require special and compassionate attention to reduce traumatic experiences [24].

## 5 Limitations

While our study had several strengths, there are also limitations. Participants were recruited from various regions across the country; however, the sample selection was not random. The convenience sample compounded the problems with a limited sample size (some cells were empty) in terms of difficulty supporting a more complex analysis. In other words, the number of covariates that could be included in the logistic regression model was limited by sample size. Drawing definitive conclusions about the quality of care in specific jurisdictions or specific subpopulations is not possible.

Inviting participants to recall and report retrospectively on childbirth experiences spanning 10 years had implications for our analyses. We attempted to control for temporal variations in care practices, such as during and after COVID‐19 lockdowns, by including yearly time trends in the analysis. Nonetheless, these adjustments would not comprehensively address differences in recall, the disparities in care delivery across different geographical locations, or changes in provincial healthcare systems over time. When conceptualizing our analysis, we intended to include the effect of parity as it related to the respectful care of families experiencing loss. The size and nature of our sample (retrospective recall) and missing data precluded those efforts. Further research in this area could explore whether increased parity predicts the type of experiences families have with perinatal loss. Some confidence intervals in the multivariate logistic regression analysis were quite broad, which can suggest unstable estimates.

The small sample size and limited data necessitated the grouping of the Indigenous and racialized subpopulations. By grouping these subpopulations, our analysis obscured their unique life struggles and experiences. While odds ratios provide insights into important data associations, they do not capture the diverse sociocultural backgrounds and historical contexts inherent within or across these subpopulations. The parameters of our study required the aggregation of sexual minority subpopulations to achieve adequate cell sizes for analysis, which precluded identification of disparities in perinatal experiences previously reported by sexual minority sub‐populations [25]. It is important that future research studies gather more nuanced data and include population‐specific researchers to address the specific challenges that these subpopulations face with perinatal loss.

## 6 Implications for Practice

Our findings have important implications for healthcare practitioners. They suggest that, given our small sample of participants, miscarriage experiences are handled less compassionately than for individuals experiencing late losses (late second trimester, stillbirth, and neonatal death). Lack of acknowledgement at the time of the loss and lack of support for the loss suggest that early losses are minimized, possibly because less time has been invested in the pregnancy and baby or because providers regard early losses as a result of ‘damaged’ babies. The review by Shorey et al. [9] examining needs and experiences of health care professionals encountering perinatal losses only focused on obstetrical and gynecological settings. Many individuals experiencing miscarriages are more likely to be seen in emergency room or laboratory settings. Providers in those settings are unlikely to have had continuing education about perinatal losses and have many competing demands from other patients. Larivière‐Bastien and colleagues reported that women experiencing miscarriages in emergency room settings lacked information from care providers at the announcement of the miscarriage, how the miscarriage would proceed, and what to expect after discharge [26]. In particular, physical symptoms, psychological consequences, and support resources were not communicated. Also, perinatal providers may well be biased towards being more empathetic towards people with a late loss. Continuing professional education and/or curricular content for all health care providers should include the perspective of childbearing people with early losses to emphasize that loss at any stage requires compassionate and respectful care.

In addition to previous reports about poor care, poorer access to care, and poorer health outcomes in general for individuals who identified as Indigenous/Black/People of Color [9], the perinatal loss experiences reported here also reflect disparities in access to high‐quality care. Such variations in provider‐patient communication are unacceptable in an inclusive society and can only be redressed through intentional individual and system changes, including in‐service education on implicit bias, anti‐racism, and accountability for harmful interactions at the health systems level.

## 7 Conclusion

In conclusion, this exploration of national data that captured the experience of diverse groups during pregnancy has provided evidence of differences in care for individuals suffering a perinatal loss, depending on the timing of the loss and the identified race/ethnicity of the individual. In particular, from participants' perspectives, miscarriage appears to be a “disenfranchised loss.”

## Conflicts of Interest

The authors declare no conflicts of interest.

## Data Availability

The data that support the findings of this study are available on request from the corresponding author. The data are not publicly available due to privacy or ethical restrictions.

## Appendix A Scale Items

1. MADM index: The seven questions used for the MADM index
  1. My provider asked me how involved in decision‐making I wanted to be.
  2. My provider told me that there are different options for my maternity care.
  3. My provider explained the advantages and disadvantages of the maternity care options.
  4. My provider helped me understand all the information.
  5. I was given enough time to thoroughly consider the different care options.
  6. I was able to choose what I considered to be the best care options.
  7. My provider respected my choices.
2. MORI Index: The seven questions used for the MORI index
  1. I felt comfortable asking questions.
  2. I felt comfortable declining care that was offered.
  3. I felt comfortable accepting the options for care that my doctor or midwife recommended.
  4. I felt pushed into accepting the options my doctor or midwife suggested.
  5. I chose the care options that I received.
  6. My personal preferences were respected.
  7. My cultural preferences were respected.
3. Six questions for the Compassionate Disclosure of Loss (CDL) index:
  The following questions are about the time when you first heard about your loss.
  1. I received the news about my baby/babies in a room where my privacy was protected.
  2. My support person (spouse, partner, family member, or friend) was present.
  3. My support person and I were given time to be on our own to come to terms with the news
  4. The healthcare provider was kind and caring to me.
  5. I felt the health care provider had the skills and training to care for people in my situation.
  6. I was given full information about options for examinations for my baby/babies, such as post‐mortem.
    (Yes, No)
    I do not remember
    Not applicable
